# T1 mapping in severe aortic stenosis: insights into LV remodeling

**DOI:** 10.1186/1532-429X-17-S1-O89

**Published:** 2015-02-03

**Authors:** Thomas A Treibel, Marianna Fontana, Patricia Reant, Maria A Espinosa, Silvia Castelletti, Anna S Herrey, Charlotte Manisty, Neil Roberts, John Yap, James Moon

**Affiliations:** 1 The Heart Hospital Imaging Centre, University College London, London, UK; 2 University of Bordeaux, CHU de Bordeaux, Bordeaux-Pessac, France; 3 Gregorio Marañon Hospital, Madrid, Spain

## Background

Aortic stenosis (AS) appears to be not just a disease of the valve, with adaptive and maladaptive myocardial remodeling playing a key role. Left ventricular (LV) remodeling in AS is characterized by cellular hypertrophy and diffuse myocardial fibrosis. Macroscopic patterns differ between patients: as hypertrophy increases, the pattern changes from normal to concentric remodeling, concentric hypertrophy and finally decompensation. T1 mapping allows non-invasive estimation of diffuse myocardial fibrosis (native T1 and extracellular volume fraction; ECV). We use this methodology to investigate and correlate macroscopic and tissue level patterns of LV remodeling in AS patients prior to aortic valve replacement (AVR) as part of a larger outcome study (RELIEF-AS Study: NCT 02174471).

## Methods

**Figure 1:**
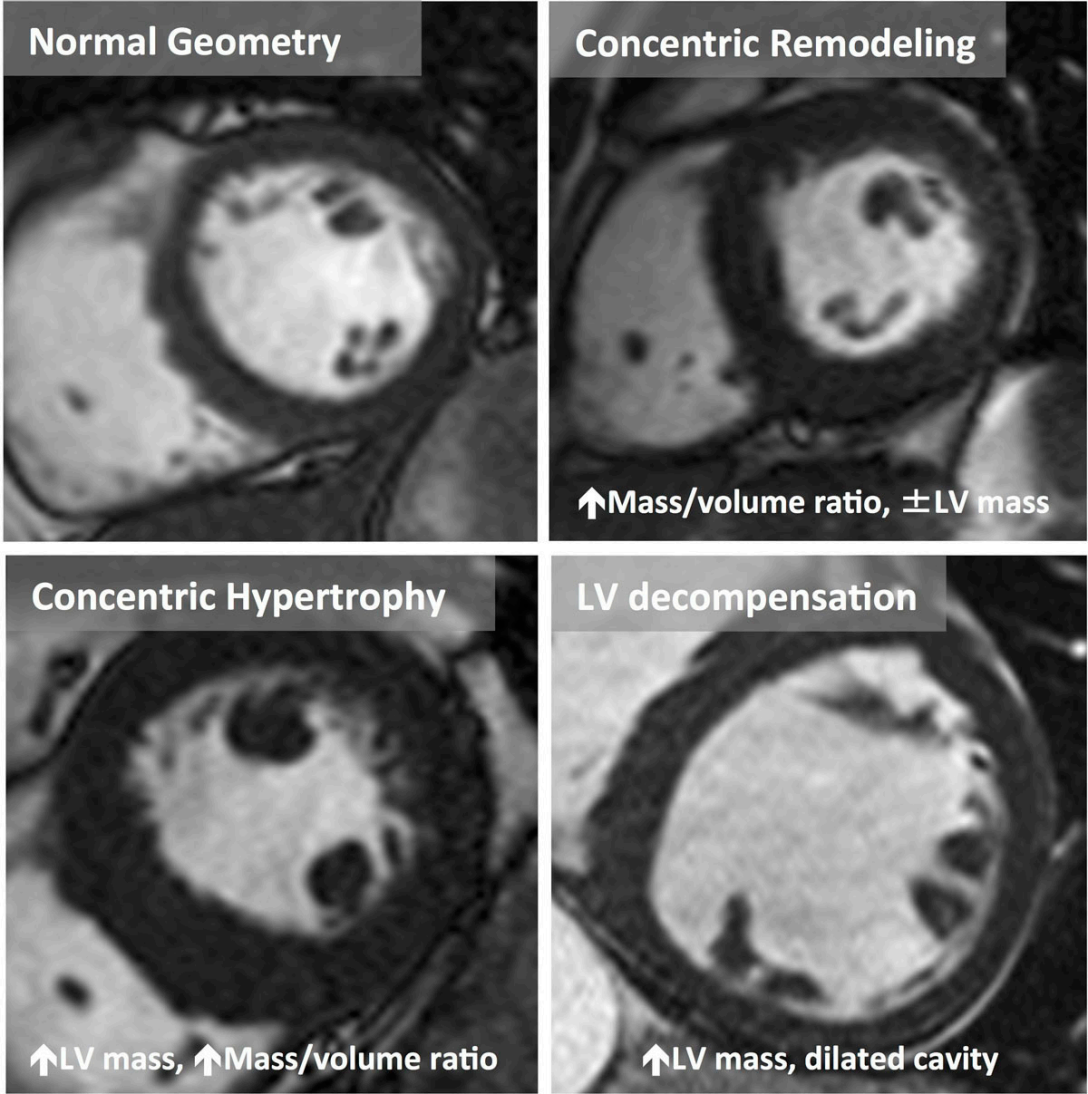
Macroscopic patterns of remodeling differ between patients: as left ventricular (LV) hypertrophy increases, the pattern changes from normal geometry to concentric remodeling (↑LV mass:volume ratio ≥1.16), concentric hypertrophy (↑LV mass and ↑LV mass:volume ratio ≥1.16) and finally decompensation (dilated LV with ↑LV mass).

135 patients (Age 70±10 years; 53% male) with severe, symptomatic AS (AVA 0.76±0.26cm^2^; Vmax 4.3±0.6m/sec) were recruited prior to AVR. CMR with T1 mapping using ShMOLLI was performed, in addition to the standard pre-operative echocardiographic assessment. LV remodeling was categorized into the 4 patterns (above, Figure 1), defined by LV mass index and the mass:volume ratio as previously described (Dweck MR JCMR 2012).

## Results

Patterns of macroscopic LV remodeling were different between genders (Table 1): normal geometry (16% of total; 81% female), concentric remodeling (34% of total; 76% female) concentric hypertrophy (62% of total, 69% male) and decompensation (23% of total; 78% male).

**Table 1:** Characteristics of patients with different patterns of remodeling.

	Normal Geometry	Concentric Remodeling	Concentric Hypertrophy	Decompensation	p-value
Number	16	34	62	23	

Male Sex, %	19	24	69	78	<0.01*

Age, years	72±9	72±8	70±11	67±10	0.22

Native T1, ms	976±37	971±33	988±38	1002±42	0.02*

ECV, %	28±3	27±2	28±3	30±2	0.03*

iAVA, cm2/m2	0.4±0.2	0.4±0.1	0.4±0.1	0.4±0.1	0.75

Vmax, m/sec	4.2±0.4	4.2±0.7	4.5±0.6	4.2±0.6	0.13

Zva index	3.6±0.5	4.8±1.0	3.7±0.8	3.6±1.3	<0.01*

NT-pro-BNP	61±71	83±120	151±152	437±433	<0.01*

Native T1 and ECV increased with more abnormal patterns of remodeling (ANOVA, *p*=*0.016* and *p*=*0.028*; Table 1), however T1 and ECV levels were gender independent (male vs female 982±37ms vs 989±39ms, *p*=*0.3;* ECV 28±3% vs 28±3%, *p*=*0.7*)*.*

Native T1 and ECV correlated with NT-pro-BNP (R=0.51 and R=0.48, *p*<*0.001*), although native T1 was a better univariate predictor (R=0.45, *p*<*0.001* vs R=0.33, *p*<*0.001*). On multivariant analysis, only LVEF, creatinine and native T1 (in order of importance) predicted NT-pro-BNP level: (R^2^=0.75, *p*<*0.001*); neither LV mass, EDV, left atrial area nor markers of afterload, diastolic function or AS severity were predictors.

## Conclusions

Patients with severe AS remodel differently and the patterns of remodeling appear to be influenced by gender with males more likely to have a hypertrophic response. Both native T1 and ECV track the prognostic markers NT-pro-BNP, with native T1 mapping the better predictor. Native T1 adds prognostic information over traditional echocardiographic markers of AS, suggesting that both macroscopic and microscopic changes contribute to the assessment of AS. We await 1-year post-AVR data from the RELIEF-AS study (due next year).

## Funding

This project is supported by a doctoral research fellowship by the National Institute of Health Research (NIHR-DRF-2013-06-102).

